# Preparation of a Specific ssDNA Aptamer for Brevetoxin-2 Using SELEX

**DOI:** 10.1155/2016/9241860

**Published:** 2016-12-12

**Authors:** Rui-Yun Tian, Chao Lin, Shi-Yu Yu, Sheng Gong, Pan Hu, Yan-Song Li, Zong-Cheng Wu, Yang Gao, Yu Zhou, Zeng-Shan Liu, Hong-Lin Ren, Shi-Ying Lu

**Affiliations:** ^1^Key Laboratory of Zoonosis Research, Ministry of Education, Institute of Zoonosis, College of Veterinary Medicine, Jilin University, Changchun 130062, China; ^2^Emergency Department, The Eastern Division, The First Hospital of Jilin University, Changchun 130062, China; ^3^Fuqing Entry-Exit Inspection and Quarantine Bureau, Port District, Qingrong Road, Fuqing, Fujian 350300, China

## Abstract

The existing assays for detecting brevetoxin (BTX) depend on expensive equipment with a professional operator or on an antibody with limited stability, which requires complex processes, a high cost, and a considerable amount of time. The development of an alternative detection probe is another promising research direction. This paper reports the use of aptamers binding to BTX-2 in an analytical assay using the systematic evolution of ligands by exponential enrichment (SELEX). After 12 rounds of selection, the secondary structures of 25 sequences were predicted. Compared to other aptamers, Bap5 has relatively high affinity with the lowest dissociation constant of 4.83 *μ*M, and IC_50_ is 73.81 ng mL^−1^. A good linear regression formula of *y* = 30.688*x* − 7.329 with a coefficient correlation of *R* ^2^ = 0.9798 was obtained using a biotin-avidin ELISA. Moreover, there is no cross-reaction with the detected marine toxins, except for BTX-2. Thus, Bap5 has potential to detect BTX-2 in shellfish in the future as a substitute for the recognition probe.

## 1. Introduction

Red tides have bloomed more frequently due to the increase in marine pollution, followed by the sprouting of a significant quantity of harmful algae [1] and the production of metabolic products from marine biotoxins, including diarrhetic shellfish poison (DSP), paralytic shellfish poison (PSP), neurotoxic shellfish poison (NSP), and amnesic shellfish poison (ASP) [2, 3]. Brevetoxins, well-known as typical NSP toxins, are initially located intracellularly in* Karenia brevis*, but the toxins become more widely available in the environment after the cells lyse or die [4]. The toxins are also transferred through the food chain and accumulated in shellfish, fish, and other species. The persistence of the toxins in seawater, sediments, and seagrass epiphytes results in chronic or sustained exposures that can damage the health of marine mammals and other aquatic invertebrates and even cause massive fish killing [5–8]. NSP toxins activate voltage-sensitive sodium channels, causing health issues in humans and animals. When humans or animals digest contaminated filter-feeding animals or swallow the seawater during blooms of* Karenia brevis*, they will experience the characteristic symptoms of NSP, such as paresthesia (tingling), reversal of hot-cold temperature sensation, myalgia (muscle pain), vertigo, ataxia (loss of coordination), abdominal pain, nausea, diarrhea, headache, bradycardia (slow heart rate), dilated pupils, and respiratory distress [5, 9].

Based on their individual structures, NSP toxins consist of at least 13 different components and have more complex structures, including brevetoxin A (BTX-A) and brevetoxin B (BTX-B) [10]. The primary component of BTX-B,* Ptychodiscus* brevetoxin-2 (BTX-2), is the most common neurotoxic shellfish poison and has attracted more attention because of the red tide event in Florida that resulted in widespread fish death [7]. BTX-2 is a fat-soluble polyether compound [11]. The minimum lethal dose in mice is 0.25 mg kg^−1^, and LD_50_ (lethal dose, 50%) is 200 mg kg^−1^. Therefore, many researchers have continued to explore sensitive and convenient methods for detecting the toxic substance. Currently, the available detection methods include solid-phase extraction (SPE) for qualitative analysis [12], high-performance liquid chromatography (HPLC) [13], liquid chromatography-mass spectrometry (LC-MS) [14], radioimmunoassay [4], electrochemiluminescence-based immunoassay [15], thin-layer chromatography [16], and immunological methods, such as enzyme-linked immunosorbent assay (ELISA), which utilizes antibodies to quantify the BTX-2 levels [17]. The disadvantages of these assays are the expensive equipment, which require professional staff, the high cost of antibodies with limited stability, and the special storage conditions required for the immunological assay. Therefore, the development of an alternative probe for the detection methods that rapidly, cost-effectively, and sensitively monitors BTX in shellfish and seawater is a pressing need.

Recently, aptamers, oligonucleotides such as single-stranded DNA (ssDNA) or RNA [18, 19], have attracted increasing attention as the sensing elements in biosensors used to detect many types of molecules, such as proteins, metals, polypeptides and small molecules [20]. Aptamers have all of the advantages of antibodies along with the unique advantages of higher specificities and affinities for the target and can be synthesized, chemically modified, easily stored, denatured, and renatured [21]. Aptamers are usually obtained via an in vitro process called systematic evolution of ligands by exponential enrichment (SELEX), which was first reported in 1990 [22]. Since then, many articles have described the use of SELEX to select aptamers targeting many types of substances, particularly the aptamers targeting and detecting toxins in recent years, such as saxitoxin [23, 24], okadaic acid [25, 26], ochratoxin A [27–30], and the recent report of BTX-2 in 2015 [31]. Based on these advantages, aptamers are emerging as novel capturing agents and recognition receptors in biosensor applications. In this study, we report the development of DNA aptamers that are able to bind BTX-2 with an dissociation constant of 4.83 *μ*M, an IC_50_ value of 73.81 ng mL^−1^, and selectivity for BTX only. The results presented in this study report a novel, alternative analytical probe for the development of an NSP toxin immunological assay. These results will contribute to the establishment of an experimental protocol for a relatively simple, affordable, and sensitive laboratory method for the detection of BTX in shellfish products and seawater.

## 2. Materials and Methods

### 2.1. Apparatus and Reagents

The BTX-2 and saxitoxin (STX) standards (purity ≥ 98%) were purchased from ZEN-U Biotechnology Co. Ltd. (Tai Wan, China). The okadaic acid (OA) and domoic acid (DA) standards were purchased from ALEXIS® Biochemicals. The monoclonal antibody against BTX-2 was prepared in our lab (*Ka* value is 0.82 × 10^9^ M^−1^) and stored at −80°C [32]. Bovine serum albumin (BSA) and ovalbumin (OVA) were purchased from the Beijing Dingguo Biotechnology Development Center (Beijing, China). The target BTX-2-BSA and BTX-2-OVA conjugates were prepared using a previously described method [32]. The DNA mate, Taq DNA polymerase, PMD-18T, and DL2000 marker were purchased from Takara Biotechnology Co., Ltd. (Dalian, China). The streptavidin-peroxidase complex was purchased from Beijing Biosynthesis Biotechnology Co., Ltd. (Beijing, China). Sterile ultrapure water was obtained from a Milli-Q water purification system (Millipore, USA). All of the polymerase chain reactions (PCRs) were conducted in a GeneAmp PCR system 9600 (Applied Perkin-Elmer, USA). The standard 96-well ELISA plates (U-bottom) were purchased from Costar (NY, USA). Absorbance was recorded using an Epoch Microvolume Spectrophotometer system (BioTek Instruments, Inc., USA). The other reagents used in this study were of analytical grade.

### 2.2. The ssDNA Library and Primer Synthesis

The initial ssDNA library used for SELEX and the homologous primers were synthesized by the Shanghai Sangon Biological Engineering Technology & Services Company (Shanghai, China). The sequences included in the ssDNA library were 85 nucleotides in length, with a central region of 40 random nucleotides flanked by two primer-binding sites for PCR and cloning, as follows: 5′-GAGGCAGCACTTCACACGATCTG-N40-CTGCGTAATGACTGTAGTGATG-3′. Primer 1 (5′-GAGGCAGCACTTCACACGATCTG-3′) and primer 2 (5′-CATCACTACAGTCATTACGCAG-3′) were used for amplification and cloning. The biotin-modified primer (5′-biotin-GAGGCAGCACTTCACACGAT-3′) was also synthesized for use in the binding assays. All of the oligonucleotides were dissolved in TE buffer (30 mM Tris-Cl, 1 mM EDTA, pH 8.0) and stored at −20°C until further use.

### 2.3. Preparation of the Target Conjugates

The target BTX-2-BSA and BTX-2-OVA conjugates were prepared using a previously described method [32]. The basic procedure is described below. A 10-fold molar excess of succinic anhydride solubilized in 2.5 mL of anhydrous pyridine was added to 2 mg of crystalline BTX-2. After a 6 h incubation at 65°C, the solvent was evaporated under a stream of nitrogen and the residue was reacted with a 10-fold molar excess of tributylamine and isobutyl chlorocarbonate as 1/10 dilutions in dry peroxide-free dioxin for 30 min at room temperature. Then, the carrier proteins BSA and OVA (molar ratio of hapten/carrier: 50 : 1) were added and incubated for 30 min at room temperature; these carrier proteins were used for the target (BTX-2-BSA) of SELEX and detection antigen (BTX-2-OVA), respectively. The complex was recovered by acetone precipitation, resuspended in 0.5 mL of distilled water, filter-sterilized (0.22 *μ*m), dispensed into sterile tubes, freeze-dried overnight, and stored at −20°C until use.

### 2.4. Selection of the Aptamer

**Scheme 1:**
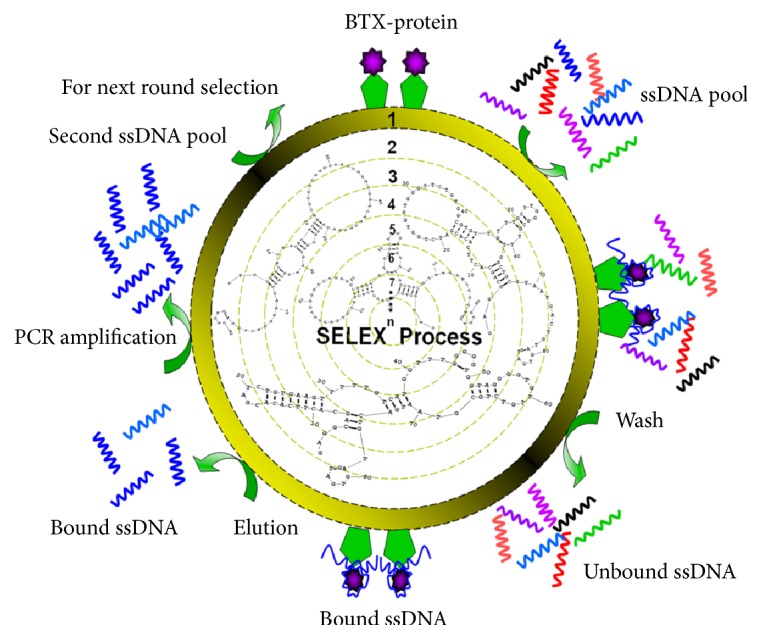
Schematic representation of the SELEX process used to select the aptamer.

The typical SELEX procedure is an iterative process in which the sequences that specifically bind to the target are selected from highly diverse synthetic nucleic acid libraries in several rounds. The principle for SELEX is shown in Scheme 1. In the selection process, the BTX-2-BSA precoated microwell plate was used to screen for specific aptamers that bound to the target toxin BTX-2. The random ssDNA pool was incubated with the target, and, then, the separated sequences were amplified to generate an enriched secondary ssDNA pool for the next round of screening. After many rounds of screening, the ssDNA sequences with a high affinity for the target were obtained.

After each selection round, the general ssDNA library was amplified using the following two types of PCR: the symmetric PCR method for producing dsDNA and the asymmetric PCR method for preparing ssDNA. The random ssDNA library and the amplified ssDNA pool obtained in each round of selection were dissolved in TE buffer and then diluted with the SHCMK binding buffer (20 mM Hepes, 120 mM sodium chloride, 5 mM potassium chloride, 1 mM calcium chloride, and 1 mM magnesium chloride). In the first round, 12 *μ*L of ssDNA (100 nM) was added to 800 *μ*L of SHCMK binding buffer, followed by denaturation at 96°C for 10 min and cooling on ice for 10 min to prevent misfolding of the ssDNA before it bound to the toxin. The preconditioning ssDNA library (100 *μ*L per well) was added to 8 wells of a microtiter plate, on which 1 *μ*g mL^−1^ BTX-2-BSA was immobilized (100 *μ*L per well). The mixture was incubated for 5 h at 37°C in the dark. Then, the liquids were decanted, and the microwell plate was washed three times with SHCMK washing buffer (SHCMK binding buffer containing 0.05% Tween-20, 220 *μ*L per well) for 1 min with gentle shaking to separate the unbound sequences from the bound sequences. The free ssDNA was discarded, and the desired ssDNA remained bound to the immobilized toxin conjugates. Elution buffer (20 mM Tris-HCl, 4 mM guanidine thiocyanate, and 1 mM DL-dithiothreitol, pH 8.3) was added to the wells (100 *μ*L per well) and incubated at 80°C for 20 min to elute the ssDNA. Phenol, chloroform, and isoamyl alcohol (25 : 24 : 1) were used to purify the ssDNA. The ssDNA was precipitated by adding dehydrated alcohol that had been precooled to −20°C and then centrifuging at 12,000*g* for 5 min to obtain the ssDNA targeting BTX-2-BSA. Finally, the precipitated ssDNA was dissolved in 20 *μ*L of TE buffer and stored at −20°C until the next round. The secondary ssDNA library was prepared using the asymmetric PCR method. Eight percent urea-polyacrylamide gel electrophoresis and silver staining were used to identify the ssDNA; silver staining was employed after electrophoresis because of its high sensitivity compared with ethidium bromide (EB) or Coomassie Brilliant Blue staining.

### 2.5. Amplification and Purification of the ssDNA

The eluted and concentrated ssDNA molecules were amplified using symmetric and asymmetric PCR. The symmetric PCR reaction is designed to obtain double-stranded DNA as the template for asymmetric PCR. The reaction system for symmetric PCR contained 20 *μ*M upstream primer, 20 *μ*M downstream primer, 10 mM dNTP mixture, 5 *μ*L of 10x PCR buffer (100 mM Tris-HCl pH 8.3, 500 mM KCl, and 15 mM MgCl_2_), 2.5 ng of ssDNA template, the concentration of which was detected using BioTek Instruments in the following screen, 1.25 U of Taq polymerase, and sterile distilled water in a total volume of 50 *μ*L. The asymmetric PCR reaction was the same as the symmetric PCR reaction, except that the ratios of the upstream and downstream primers were increased 100-fold. The PCR cycling conditions consisted of an initial denaturation at 96°C for 2 min; 25 cycles of 96°C for 30 s, 64°C for 30 s, and 72°C for 30 s; and a final extension at 72°C for 5 min. The ssDNA was amplified from asymmetric PCR using the same conditions, but with an annealing temperature of 59°C for 20 cycles. The 5′-biotin-labeled products of the asymmetric PCR were used for an affinity analysis in each round. The dsDNA obtained from the round with the best affinity was used for cloning after it was purified according to the instructions of the Poly-Gel DNA Extraction Kit (OMEGA) [33].

### 2.6. Cloning and Sequencing Analysis

The purified and highly concentrated oligonucleotide from the best round of selection was cloned into a pMD18-T vector and transformed into competent* Escherichia coli* DH5*α* cells. Positive colonies were verified by PCR. Complete sequencing of the samples was performed by Shanghai Sangon Biotechnology. After cloning and sequencing, the lowest free-energy shapes and secondary structures were predicted and the sequences were analyzed using the free-energy minimization algorithm in the DNAMAN and Mfold programs.

### 2.7. Identification of Specificity and Affinity

An indirect ELISA was performed after each round of selection to test the process of aptamer selection and evaluate the binding affinity of the selected aptamers. For this purpose, 100 *μ*L of 0.5 *μ*g mL^−1^ BTX-2-OVA (1, 0.5 and 0.25 *μ*g mL^−1^ BTX-2-OVA were coated to determine the *Ka* value) diluted with coating buffer (0.05 M sodium carbonate, pH 9.6) was immobilized on the surface of the microwells and incubated at 4°C overnight. The plate was washed three times with the SHCMK cleaning solution (220 *μ*L per well) followed by incubation with 100 *μ*L of 5% evaporated skim milk at 37°C for 2 h. Increasing concentrations (0–20 nM) of the 5′-biotin-labeled aptamer (100 *μ*L per well) were added to the washed plate and incubated at 37°C for 1 h. After another washing step with the SHCMK cleaning solution, 100 *μ*L of diluted horseradish peroxidase-conjugated streptavidin (1 : 1,000) in PBS containing 0.1% BSA was added to each well and incubated for 1 h at 37°C. After four gentle washes, 100 *μ*L aliquots of the* o*-phenylenediamine/H_2_O_2_ solution in 0.05 M citrate–phosphate buffer (pH 5.0) were added to each well. The reaction was stopped by the addition of 50 *μ*L of a 2 mM H_2_SO_4_ solution to each well and a 10 min incubation at room temperature. The optical density was measured at 492 nm (OD_492_) on an Epoch Microvolume Spectrophotometer system. The *Kd* value was calculated using the equation *Kd* = 1/*Ka* = (*n*[Ap′]*t* − [Ap]*t*)/(*n* − 1). In this equation, *n* is the concentration ratio of plates coated with two different concentrations of BTX-2-OVA in one group and [Ap′] and [Ap] are the concentrations (mol L^−1^) of the aptamer corresponding to 50% of the maximum absorbance values of the plates coated with the two different concentrations of BTX-2-OVA. The average is the affinity constant. The available marine biotoxins BTX, STX, OA, and DA were tested for cross-reactivity using the same affinity measurement methods to evaluate the specificity of the aptamer.

### 2.8. Competitive Inhibitory Activity

An indirect competitive ELISA was performed to determine the inhibitory activity of the selected aptamer Bap5. The BTX-2-OVA conjugate was diluted with 0.05 M bicarbonate buffer (pH 9.6), added to a microtiter plate at a concentration of 0.5 *μ*g mL^−1^, and incubated at 4°C overnight. The plate was washed three times with the SHCMK cleaning solution (220 *μ*L per well) followed by incubation with 100 *μ*L of 5% evaporated skim milk at 37°C for 2 h. After 3 washes, equal volumes (50 *μ*L per well) of solutions of the BTX-2 standard (200, 100, 50, 25, 6.25, 3.125, and 0 ng mL^−1^) diluted in 10 mM PBS (pH 7.4) were mixed with 5 nM biotin-labeled ssDNA diluted in the SHCMK binding buffer in each well and incubated at 37°C for 1 h. The subsequent steps were performed in the same manner as those described in Identification of Specificity and Affinity. The calibration curve was obtained, with the percent inhibition [(*N* − *S*)/*N* × 100%] on the *y*-axis and the log BTX-2 concentration on the *x*-axis. *N* is the OD_492_ value when the toxin standard is not present in the detected sample (control), and *S* is the OD_492_ value observed when there is a difference in the concentration of the standard in the detected sample.

## 3. Results and Discussion

### 3.1. Selection of the Aptamer

**Figure 1:**
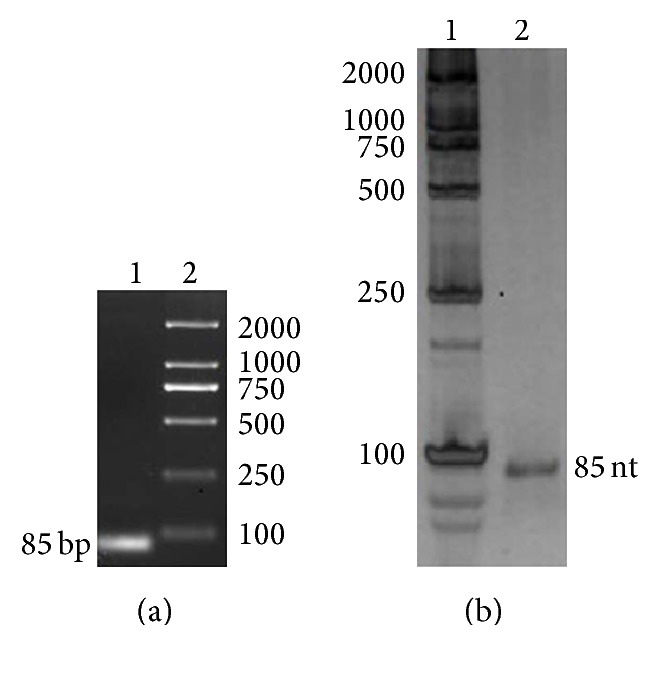
Identification of dsDNA and ssDNA using electrophoresis. (a) 2% sepharose gel electrophoresis of the 85-bp dsDNA amplified by symmetric PCR. Lane 1 is the 85-bp dsDNA, and lane 2 is the DL2000 DNA Marker; (b) 8% denaturing urea gel electrophoresis of ssDNA. Lane 1 is the DL2000 DNA Marker, and lane 2 is the 85-nt ssDNA amplified by asymmetric PCR.

Aptamers against BTX-2 were obtained using the SELEX method in vitro. The specificity and quantity of the ssDNA and the dsDNA were significantly affected by the PCR reaction system and the thermocycler conditions. The PCR conditions were optimized to obtain a high volume and specific ssDNA. Gradient temperature PCR was chosen as the optimization method to avoid nonspecific amplification. Using this method, 64°C and 25 cycles were the optimal settings for the production of a high content of dsDNA with sufficient purity (data not shown). The generation of high purity ssDNA is a key factor in obtaining aptamers with high affinity and selectivity. In this study, asymmetric PCR was used to generate ssDNA with a 1 : 10 ratio of forward primer and reverse primer. Then, 2% sepharose gel electrophoresis was used to identify the amplified dsDNA (Figure 1(a)), whereas 8% urea-polyacrylamide gel electrophoresis was used to analyze the ssDNA (Figure 1(b)). As shown in Figures 1(a) and 1(b), the target bands indicated the correct dsDNA and ssDNA products, without dimers and nonspecific DNA. The highly specific ssDNA provided an excellent secondary library for the subsequent screen.

**Figure 2:**
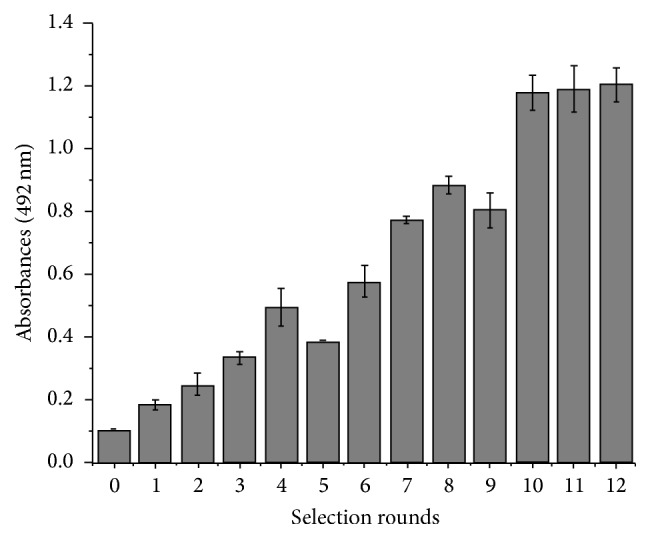
Binding of the ssDNA library to BTX-2-BSA during the 12 SELEX rounds.

The counterselection process was performed after every 4 rounds of selection using the immobilized BTX-2-OVA as the alternative conjugate to enhance the selectivity and specificity of the aptamer. The negative selection steps can remove the sequences that bind to BSA and the plates or molecules that may be bound nonspecifically. After the 12 rounds of selection, an indirect biotin ELISA was used to identify the binding capacity of selected aptamers. As shown in Figure 2, the absorbance values recorded at 492 nm gradually increased from 0.108 in the first round to 0.537 in round 4, 0.907 in round 9, and 1.256 in round 12. Thus, the binding capacity between the aptamer and BTX-2 increased. Therefore, the specific aptamer library was gradually enriched. In contrast, the absorbance values obtained in the 5th and 9th rounds were slightly decreased. Thus, the nonspecific aptamers targeting BSA, the plates, and the other materials had been removed during the negative selection. However, the absorbance increased and reached its maximum at the 12th round and then stabilized. Therefore, as the binding reaches a plateau, the affinity reaches saturation and the enriched high affinity aptamers account for the majority of the ssDNA library. After selection, we amplified the dsDNA from the 12th round using the symmetric PCR process described above and purified the DNA using an AxyPrep DNA Gel Extraction Kit. The purified and highly concentrated oligonucleotides were cloned into the pMD18-T vector and transformed into competent* Escherichia coli* DH5*α* cells, and their inserts were confirmed by PCR and sequenced prior to the subsequent experiments.

### 3.2. Secondary Structure Predictions

**Figure 3:**
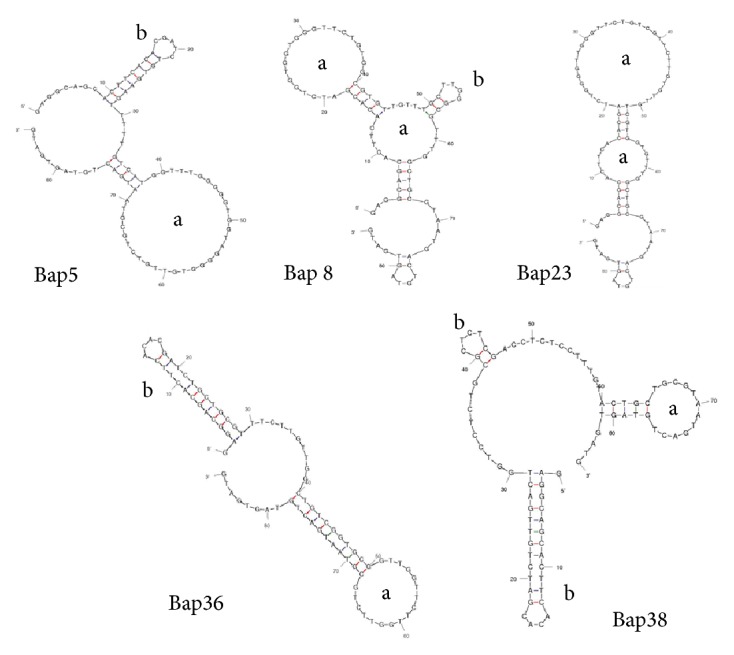
Secondary structures of the candidate aptamers predicted by Mfold.

After cloning and sequencing, the secondary structures of the 25 sequences were further predicted and analyzed using the DNAMAN and Mfold programs. The lengths of most of the sequences were the same as those in the anticipated library. Two repeated sequences were enriched in guanine, and the other sequences reached greater than 70% identity. Based on this result, the ssDNA library was enriched after 12 rounds of SELEX. Generally, oligonucleotides fold into various secondary structures, such as stem-loop, pseudoknot, hairpin, pocket, and even G-quadruplexes, which act as the binding motifs or the region responsible for biological activity. The most likely minimum energy secondary structures of the selected aptamers were analyzed with the Mfold program to predict the structure or the aptamer that bound to the target toxin [33]. In this study, the secondary structures of most of the obtained aptamers were typical stem-loop (a) and hairpin-loop (b) structures; Figure 3 shows some of the aptamers. The analysis of the secondary structures clearly showed that the aptamers formed the loop structure using the random region, and the G-rich sequence likely forms a G-quartet structure. We predict that the structures of all the aptamers might play particularly crucial roles in binding to the target and are the predicted target-binding site.

The letters (a) and (b) indicate the stem-loop and the hairpin-loop, which is the possible region in the aptamer that binds to the BTX-2 toxin.

### 3.3. Binding Affinity and Specificity

**Figure 4:**
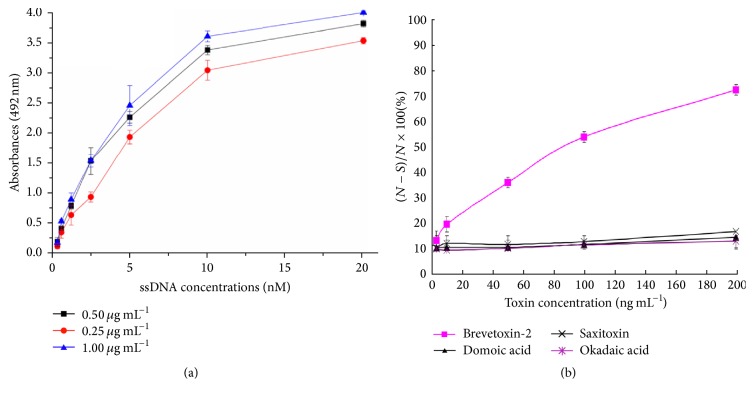
Binding activity and cross-reactivity of Bap5. (a) The *Kd* value of the Bap5 aptamer was measured using different BTX-2-OVA coating concentrations (1, 0.5, and 0.25 *μ*g mL^−1^) to estimate the dissociation constant (*Kd* = 1/*Ka* = (*n*[Ap′]*t* − [Ap]*t*)/(*n* − 1)). The *x*-axis represents the aptamer concentration (ng mL^−1^), whereas the *y*-axis represents the absorbance at 492 nm. (b) Plots from the ELISA experiments used to investigate the specificity of the Bap5 aptamer for BTX-2 and other toxins. The calibration curve was obtained with the inhibition percent [(*N* − *S*)/*N* × 100%] shown on the *y*-axis and the concentration of toxins on the *x*-axis. *N* is the OD_492_ value when the toxin standard is not present in the detected sample (control), and *S* is the OD_492_ value when there is a difference in the concentration of the standard in the detected sample. All the data represent the means ± SD (*n* = 3) of three replicates.

An indirect biotin ELISA was performed using concentrations of BTX-2-OVA from 0.25–1 *μ*g mL^−1^as the coated substance to evaluate the dissociation constant and specificity of the binding of the candidate aptamers to BTX-2. In addition, the aptamer concentration was diluted in a gradient from 0.625 nM to 20 nM. The *Kd* value was calculated using the equation *Kd* = 1/*Ka* = (*n*[Ap′]*t* − [Ap]*t*)/(*n* − 1). The curve for the Bap5 aptamer is shown in Figure 4(a). The *Kd* value for the binding between the Bap5 aptamer and BTX-2 was 4.83 *μ*M, whereas the values for Bap 8, Bap 23, Bap 36, and Bap 38 were 9.80 *μ*M, 7.25 *μ*M, 10.47 *μ*M, and 10.55 *μ*M, respectively. Among the five selected aptamers, Bap5 has the lowest *Kd* value and a high binding affinity for BTX-2, similar to a previous report [28]. The *Kd* values for Bap 8, Bap 23, Bap 36, and Bap 38 were relatively high and were thus considered to have weak binding capacity and were discarded from further experiments. Cross-reactivity was assessed using the available marine toxins DA, STX, and OA under the same conditions to further investigate the specificity of the Bap 5 aptamer for BTX-2. As shown in Figure 4(b), Bap 5 had an overwhelmingly positive response to BTX-2 compared with the weak responses to the other toxins. Because the other BTXs are not available, the cross-reactivity with these toxins must be assayed in a future study.

### 3.4. Competitive Inhibitory Activity

**Figure 5:**
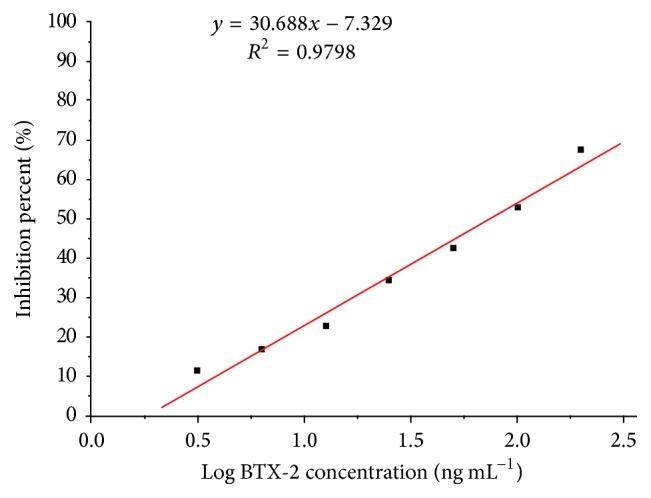
Plot of the indirect competitive ELISA of the BTX-2 aptamer Bap5. The calibration curve was obtained with the inhibition percent [(*N* − *S*)/*N* × 100%] on the *y*-axis and the log concentration of BTX-2 on the *x*-axis. The *N* and *S* values were the OD_492_ of the control and the sample, respectively. As the concentration of the BTX-2 standard increased (ranging from 3.125 to 200 ng mL^−1^), the inhibition ratio ranged from 11.67% to 67.3%.

An indirect competitive ELISA based on the Bap5 aptamer was performed to assay the inhibitory activity of this aptamer. A good linear regression formula, of *y* = 30.688*x* − 7.329, with a coefficient correlation of *R* ^2^ = 0.9798 was obtained (Figure 5). The standard curve was linear over the range of 3.125–200 ng mL^−1^ with an IC_50_ (half maximal inhibitory concentration of BTX-2) value of 73.81 ng mL^−1^ and a detection limit of 3.125 ng mL^−1^, which was greater than the sensitivity of the NSP ELISA kit (Abraxis, America, Product No. PN520026) and the value reported in our previous study (IC_50_ is 5.3 ng mL^−1^) using the monoclonal antibody [32] but less than the current European Union and Codex Alimentarius Commission regulatory limit (800 *μ*g/kg shellfish meat). Notably, the use of aptamers as a substitute for the antibody has many specific advantages because the aptamers can be synthesized, chemically modified, easily stored, denatured, and renatured. Therefore, we anticipate that the aptamer screened in this study will be applied as another recognition probe in an aptamer-based detection assay for assessing the BTX levels in contaminated seafood.

## 4. Conclusion

In this study, aptamers targeting BTX-2 were successfully obtained via 12 rounds of SELEX selection. Compared to other aptamers, Bap5 showed relatively high affinity with a low dissociation constant of 4.83 *μ*M and the best IC_50_ value of 73.81 ng mL^−1^. The Bap5 aptamer did not show cross-reactivity with the other detected marine toxins. Based on the high sensitivity and specificity of Bap5, it may be used as an alternative analytical probe for the development of a detection assay for BTX-2. Future studies should aim to develop an assay based on the obtained aptamer for the detection of BTX-2 in marine food samples to protect consumer safety and promote the development of aquaculture.
